# The fitness connection of antibiotic resistance

**DOI:** 10.3389/fmicb.2025.1556656

**Published:** 2025-04-10

**Authors:** Miklos Fuzi

**Affiliations:** Independent Researcher, Seattle, WA, United States

**Keywords:** fluoroquinolones, resistance, clones, energy saving schemes, fitness

## Abstract

More than three decades ago multidrug-resistant (MDR) clones of the pathogens: *Staphylococcus aureus, Escherichia coli, Klebsiella pneumoniae, Clostridioides difficile, Enterococcus faecium, Pseudomonas aeruginosa and Acinetobacter baumannii* have started to disseminate across wide geographical areas. A characteristic feature of all these MDR lineages is the carriage of some mutations in the quinolone resistance-determining regions (QRDRs) of DNA gyrase and topoisomerase IV which besides conferring resistance to fluoroquinolones are associated with a fitness benefit. Several lines of evidence strongly suggest that extra fitness conferred by these mutations facilitated the dissemination of the international MDR lineages. MDR pathogens require extra energy to cover the fitness cost conferred by the excess antibiotic resistance gene cargo. However, extra energy generated by upgraded metabolic activity was demonstrated to increase the uptake of antibiotics enhancing susceptibility. Accordingly, MDR bacteria need additional positive fitness schemes which, similarly to the QRDR advantage, will not compromise resistance. Some of these, not clone-specific effects are large genomes, the carriage of low-cost plasmids, the transfer of plasmid genes to the chromosome, the application of weak promoters in integrons and various techniques for the economic control of the activity of the integrase enzyme including a highly sophisticated system in *A. baumannii.* These impacts – among others – will confer a fitness advantage promoting the spread of MDR pathogens. However, even the potential of extra fitness generated by the combined effect of various schemes is not without limit and virulence-related genes or less relevant antibiotic resistance gene cargoes will often be sacrificed to permit the acquisition of high-priority resistance determinants. Accordingly major MDR clone strains are usually less virulent than susceptible isolates. In summary, a fitness approach to the research of antibiotic resistance is very useful since the fitness status of MDR bacteria seem to profoundly impact the capacity to disseminate in the healthcare setting.

## 1 Introduction

Resistance to antibiotics in bacterial pathogens constitutes a serious challenge worldwide endangering patients mostly in the healthcare setting and resulting in considerable excess mortality. Though antibiotic resistance had appeared shortly after the introduction of the first antibiotics (Acar et al., 2012) it became a major issue in the 1990s when some genetically related groups of various pathogenic MDR bacteria started to disseminate across wide geographical swathes and have ever since remained with us in many places. Interestingly the spread of these MDR strains proved exclusively clonal and was observed primarily in a handful of pathogens: *Staphylococcus aureus, Escherichia coli, Klebsiella pneumoniae, Clostridioides difficile, Enterococcus faecium, Pseudomonas aeruginosa*, and *Acinetobacter baumannii* (Hawkey and Jones, 2009; Fuzi, 2016; Fuzi et al., 2017; Fuzi et al., 2020; Fuzi and Sokurenko, 2023). The question arises what could have triggered the dissemination of these MDR lineages?

Several lines of strong circumstantial evidence suggest a “fluoroquinolone connection.” Conspicuously, the emergence of the MDR international clones commenced in the 1990s when the consumption of fluoroquinolone type antibiotics, and primarily that of ciprofloxacin, rose substantially (Linder et al., 2005). Moreover, fluoroquinolone resistant pathogens were demonstrated to carry significantly bigger and more diverse antibiotic resistance gene cargoes than fluoroquinolone susceptible strains (Coluzzi et al., 2023). Furthermore, the onset of mutations in the quinolone resistance-determining regions (QRDRs) in *E. coli* was shown to always precede the subsequent acquisition of the excess resistance gene loads (Coluzzi et al., 2023). Among these early onset QRDR mutations the most common alterations preceding the evolution of an MDR phenotype usually affect the *gyrA* S83 and *parC* S80 residues (positions for *E. coli*) (Coluzzi et al., 2023). However, how can all of these observations be linked to the success of the major international MDR clones of human pathogenic bacteria?

## 2 An excess gene cargo involves fitness cost

MDR bacteria inescapably carry an extra cargo of genes that will provide adequate protection in an antibiotic environment like the healthcare setting. However, the carriage and expression of most of the antibiotic resistance genes are well-established to compromise fitness (Andersson and Hugnes, 2010). Moreover, many resistance-associated mutations or changes in the target molecules of antibiotics exert additional negative fitness effects (Melnyk et al., 2015; Vanacker et al., 2023). Accordingly, the successful handling of the fitness issue is crucial for MDR pathogens which need not only protection against antibiotics used in the healthcare setting but also have to retain an appropriate growth rate (fitness) permitting dissemination. Several groups emphasized that the fitness status of pathogens plays a salient role in governing dissemination in the healthcare setting (Martínez and Baquero, 2002; Shin and Ko, 2015; Bartke et al., 2021; Baquero et al., 2021; Dunn et al., 2021).

The efficient regulation of gene acquisition and the level of expression is of utmost importance not just for the reduction of fitness cost associated with resistance to antibiotics but also for retaining a proper fitness balance across the entire genome during adaptation to diverse environmental conditions (Holden and Webber, 2020; Elg et al., 2024). When bacteria need to increase the expression of a particular set of genes they will reduce the expression of some other groups of genes which are less relevant under existing conditions (Holden and Webber, 2020). This trade-off can involve both the antibiotic resistance genes and virulence genes: bacteria were observed to increase the expression of some resistance genes upon exposure to antibiotics, while, concurrently reduced the expression of virulence genes (Holden and Webber, 2020). Accordingly, bacteria to preserve fitness may become less pathogenic when showing an MDR phenotype (Holden and Webber, 2020).

These observations suggest that successful MDR bacteria should command better fitness than less prevalent MDR pathogens allowing their extensive dissemination in the healthcare setting often with the maintenance of some virulence features. The question arises why can not bacteria simply enhance their metabolic activity to cover the fitness cost conferred by the extra antibiotic resistance gene cargo? The reason is that increased metabolic activity will not only generate extra energy. The import of more nutrients will also result in a substantial rise in antibiotic uptake which will greatly enhance susceptibility (Stokes et al., 2019; Lopatkin et al., 2021; Tao et al., 2023). This is the reason why metabolic activity is well-established to be scaled back during antibiotic exposure in bacteria even though the pathogens would need extra energy (Lopatkin et al., 2021).

Consequently, we presume, that MDR bacteria should pursue alternative fitness generating/saving schemes which will not seriously compromise resistance to antibiotics. We suggest that bacteria possess/employ a variety of devices to attain this goal. The international MDR clone strains of pathogenic bacteria command a specific beneficial fitness feature. As we shall see these agents proved capable of evolving some energetically favorable QRDR mutations which besides providing resistance to fluoroquinolones confer a fitness advantage onto the isolates without compromising antibiotic resistance.

In the following the paper will provide an up-to-date analysis of the QRDR impact and, for the first time, will review several additional energetically favorable fitness effects which support the acquisition and expression of an antibiotic resistance gene cargo without enhancing susceptibility. It is supposed that the combined effect of these features govern a bacterial pathogen’s ability to economically handle an MDR phenotype and assist in preserving sufficient fitness permiting dissemination in the healthcare setting.

## 3 Two energetically favorable QRDR mutations

### 3.1 Two QRDR mutations carried by most major clone MDR pathogens

It is a characteristic feature of all the major MDR clones in various bacterial species that they carry at least one but usually both of the so-called double-serine QRDR mutations affecting the *gyrA* S83 and *parC* S80 positions in. *E. coli* or equivalent positions in other species (Fuzi et al., 2017; Fuzi et al., 2020; Fuzi and Sokurenko, 2023). The onset of these mutations, as stated above, precedes the acquisition of excess genetic material associated with resistance to diverse groups of antibiotics (Coluzzi et al., 2023). An abundance of papers demonstrated that these mutations, but especially those affecting the *gyrA* S83 position, will confer a fitness benefit onto the pathogens, measured by the growth rate under laboratory conditions. This was observed in *E. coli* (Komp Lindgren et al., 2005; Marcusson et al., 2009; Machuca et al., 2014; Huseby et al., 2017), *Salmonella Typhimurium* (Giraud et al., 2003), *Salmonella Typhi* (Baker et al., 2013), *C. difficile* (Vernon et al., 2019), and *Campylobacter jejuni* (Luo et al., 2005).

The fitness impact of the *parC* S80 mutation proved more dependent on the genetic background of the tested isolates. It was investigated in only two pathogens: *E. coli* and *Salmonella* spp. The *parC* S80 mutation was observed to confer a slight fitness cost in two studies (Marcusson et al., 2009; Huseby et al., 2017), while an additional study reported a fitness gain (Baker et al., 2013).

Similarly, the combined fitness effect of the double-serine *gyrA* S83 plus *parC* S80 mutations (positions in *E. coli*) was strongly influenced by epistasis. The double-serine mutations were linked to a small fitness cost in two studies (Marcusson et al., 2009; Huseby et al., 2017), however, a third study demonstrated a highly significant fitness benefit in a distinct *E. coli* isolate (Machuca et al., 2014). Moreover, in *P. aeruginosa* and *Burkholderia cepacia* alterations equivalent to the double QRDR mutations were shown to enhance fitness influenced also by epistasis (Pope et al., 2008; Agnello et al., 2016).

Nevertheless, the question arises why have nearly all bacterial species evolutionarily preserved the double-serine QRDR residues if they ab ovo compromise (or may compromise in a strain-specific fashion) the activity of the enzymes?

### 3.2 Common natural compounds are toxic to QRDR mutants

Interestingly the penetrance of the serine residue in positions equivalent to that of *gyrA* 83 in *E. coli* is not complete across the kingdom of bacteria. Some *Ehrlichia* spp. and most *Mycobacteria* spp. were shown to harbor non-serine residues in the equivalent positions (Maurin et al., 2001; Guillemin et al., 1998). Since *Ehrlichia* spp. virtually spend their lifetime in their insect hosts where they remain fairly protected from environmental impacts and *Mycobacteria* command a hardly penetrable cell wall, this observation may hint that the serine residue might confer protection against some naturally occurring noxa to which bacteria are exposed in the environment.

Japanese scientists showed that fluoroquinolone resistant strains of *S. aureus* carrying the double-serine mutations were all susceptible to a natural antibiotic (nybomycin, a quinolone-dione) produced by several *Streptomyces* species (Hiramatsu et al., 2012) and a very common herbal alkaloid, apigenin (Morimoto et al., 2015). Moreover, nybomycin also showed activity against fluoroquinolone resistant *Enterococci* (Hiramatsu et al., 2012). Though the effect of nybomycin was recently shown to be non-mutation specific in *E. coli* (Shiriaev et al., 2023), resistance to apigenin seems to be unequivocally linked to the *gyrA* 84 serine residue (position in *S. aureus*) (Hiramatsu et al., 2012). Moreover, a recent study has shown that multiple additional herbal flavonoids inhibit fluoroquinolone resistant *S. aureus* and *E. faecium* strains in a similar fashion (Morimoto et al., 2023). Likewise to apigenin these alkaloids are very common in nature (Sen et al., 2016). It is tempting to speculate that due to extensive exposition to herbal flavonoids and in the absence of quinolones, ancient bacteria compromised the activity of their DNA gyrase enzymes by evolving the *gyrA* S83 (*E. coli*) and equivalent residues (Hiramatsu et al., 2012).

Though apigenin was reported to be more effective against gram-positive than gram-negative bacteria (Osonga et al., 2019; Shiriaev et al., 2023), under natural conditions bacteria are exposed to the combined effect of an abundance of substances and some (perhaps many) of them could facilitate the penetration of flavonoids into the gram-negative cell wall. Obviously exposure to antibiotics constitute a much greater threat for modern bacteria in the healthcare setting than environmental noxa. Accordingly bacterial pathogens nowadays can reliably mutate their double-serine QRDR residues under the antibiotic pressure despite they being impacted by the naturally toxic compounds.

In the following chapters we are going to review both the genetic and epidemiological evidence supporting a role for both the energetically favorable QRDR mutations and some other factors in the dissemination of the international MDR clones of high priority bacterial pathogens (Table 1; Tables S1, S2). Although every human pathogenic bacterial species exposed to fluoroquinolones was impacted a review of all affected species lies beyond the scope of this paper. Similarly, the influence of slow-degrading fluoroquiunolones in the environment on the acquisition of antibiotic resistance (Frade et al., 2014; Bungau et al., 2021) can not be covered.

**Table 1:** Characteristic double-serine/threonine QRDR mutations in the international clones/lineages of various MDR pathogens (references in text).

Pathogen	Clone	Affected position in gyrA	Affected position in parC
Methicillin-resistant *Staphylococcus aureus*	ST8*, CCT5, ST22*, ST228	Ser84Leu	Ser80Phe
Multidrug-resistant *K. pneumoniae*	ST11, ST15, ST101, ST147, ST258, ST307	Ser83Phe, Ser83Ile	Ser80Ile
Multidrug-resistant *E. coli*	ST131 H30, ST1193, ST410	Ser83Ile	Ser80Ile
Multidrug-resistant Clostridioides difficile**	ribotype 027	Thr82Ile	***
Multidrug-resistant *Enterococcus faecium*	CC17 and related STs	Ser83Tyr, Ser83Arg	Ser80Ile, Ser80Arg
Multidrug-resistant Pseudomonas aeruginosa**	ST111, ST235, ST175	Thr83Ile	Ser87Leu
Multidrug-resistant *Acinetobacter baumannii*	IC1, IC2, IC3, IC4, IC5, IC6, IC7, IC8	Ser83Leu	Ser80Leu

*Some lineages of the sequence type failed to evolve the double-serine mutations. **Multiple local lineages carrying the favorable mutation(s) exist. ***Clostridioides difficile is void of the parC gene.

## 4 International MDR clone pathogens command epidemiological advantage in a fluoroquinolone environment

### 4.1 Methicillin-resistant *Staphylococcus aureus*

It was our group that demonstrated for the first time that fitness advantage commanded by the major international clones of an MDR pathogen potentially promoted their dissemination (Table 2). The global spread of the international lineages of health care-associated (HA)-MRSA about three decades ago (Nübel et al., 2011; Grundmann et al., 2014; Lawal et al., 2022) resulted in clonal shifts worldwide. In Hungary initially the ST5 and ST228 clones achieved dominance over previous minor STs (Conceição et al., 2007) what was followed by an invasion of ST22 isolates (Grundmann et al., 2014) (Table S1).

**Table 2:** Superior fitness of international MDR clone strains vis-a-vis minor clone isolates demonstrated by propagation assays.

Article	MDR pathogen	Result of fitness test
Horvath et al. (2012)	MRSA	ST5, ST22 strains transcend fluoroq. resistant ST30, ST8 minor clone isolates
Knight et al. (2012)	MRSA	CC22 strains transcend CC30, ST239 fluoroq. resistant isolates
Toth et al. (2014)	MDR *K. pneumoniae*	ST11, ST15, ST147 strains transcend fluoroq. resistant minor clone isolates
Johnson et al. (2015b)	MDR *E. coli*	ST131 H30 strains transcend minor clone isolates when exposed to fluoroq.
Vernon et al. (2019)	MDR *C. difficile*	GyrA Thre82Ile confers a fitness advantage on 027 strains of *C. difficile*

Since the epidemiological shifts were associated with a rise in the incidence of HA-MRSA in Hungary (Horvath et al., 2012; Fuzi, 2016) we investigated whether or not distinct fitness commanded by the various clones could have contributed to their success. We demonstrated that fitness costs across clones elicited by resistance to fluoroquinolones were highly diverse and could have contributed to the observed clonal replacements. Propagation assays conducted by fluoroquinolone resistant major clone strains, minor clone isolates in which resistance to ciprofloxacin was induced *in vitro* clearly showed that the major clones commanded superior fitness (Horvath et al., 2012). The significantly faster growth rate detected in the fluoroquinolone resistant major clone isolates might have accounted for both the clonal shifts and the rise in the incidence of HA-MRSA (Horvath et al., 2012). Trends in the consumption of fluoroquinolones also proved closely associated with the increase in rates for HA-MRSA in Hungary (Fuzi, 2016). Concurrently, Knight et al. (2012) investigating the fitness of strains from diverse HA-MRSA clones in the United Kingdom reported identical findings with our results. Subsequently, Holden et al. (2013) emphasized that the success of the ST22 clone was associated with the acquisition of fluoroquinolone resistance (Table S1).

Moreover, a plethora of papers have been published on clonal shifts where major clones of HA-MRSA strains (primarily CC5 and ST22) replaced diverse minor clones/STs in adult wards in the healthcare setting or at country level supporting the notion of a fluoroquinolone impact (Velazquez-Meza et al., 2004; Ma et al., 2006; Amorim et al., 2007; Conceição et al., 2007; Aires-De-Sousa et al., 2008; Knight et al., 2012; Espadinha et al., 2013; Lim et al., 2013; Lawes et al., 2015; Hsu et al., 2015; De Oliveira et al., 2019; Junaidi et al., 2023; Coombs et al., 2023) (Table S1). The findings of De Oliveira et al. (2019) are especially interesting: they observed that MRSA strains carrying the double-serine mutations replaced isolates with single-serine alterations even if they belonged to an identical sequence type. Furthermore, multiple groups reported a significant association between fluoroquinolone use and the incidence of HA-MRSA (LeBlanc et al., 2006; Couderc et al., 2014; Fuzi, 2016; Wang et al., 2019; Bolla et al., 2020; Corcione et al., 2021; Tan et al., 2022; Aldeyab et al., 2022; Chen et al., 2022; Baede et al., 2023) that obviously implies a “major-clone-effect” (Table S2).

The most important determinant of the success of the major clones of HA-MRSA (ST5, ST22) – as it was mentioned previously – should have been the characteristic carriage of the energetically favorable double-serine QRDR mutations affecting the *gyrA* S84 and *grlA* S80 residues which are mostly absent from minor lineages which often harbor additional QRDR alterations that might be associated with fitness cost (review: Fuzi et al., 2017) (Tables 1, 3).

**Table 3:** Energy saving/generating techniques/features in MDR bacteria mitigating fitness cost associated with the carriage of an antibiotic resistance gene cargo (see references in text).

Technique/feature	Mechanism	Scope
Energetically favorable QRDR mutations affecting gyrA S83 and parC S80 (*E. coli*)	Amelioration of the activities of the DNA gyrase and topoisomerase IV enzymes	Clone-specific
Genome size	Large genome mitigates fitness cost associated with an extra resistance gene cargo	Species specific
Use of attuned integrase promoters	Tailoring the expression of integron genes on both plasmids and the chromosome	Neither clone nor species specific
The use of low cost plasmids	▪ compensatory mutations	Neither clone nor species specific
	▪ moving plasmid genes to the chromosome	Neither clone nor species specific
	▪ use of attuned promoters in integrons on plasmids	Neither clone nor species specific
	▪ additional devices to be explored	
Restriction of integrase activity	▪ Silencing/elimination of the integrase gene	Neither clone nor species specific
	▪ Use of special, customized integron promoters	Species specific*
Techniques to be explored: Energy saving associated with the Management of phages Management of MGEs Management of ncRNAs Metabolic pathways not enhancing susceptibility to antibiotics Additional devices to be identified		

* Acinetobacter baumannii.

Interestingly the American ST8 clone (USA300) carrying *gyrA* S84; *grlA* S80 mutations proved capable of disseminating in US hospitals at an earlier time period when the consumption of fluoroqinolones used to be higher (Planet, 2017; Challagundla et al., 2018) and subsequently evolved into a global MDR HA-MRSA clone (Glaser et al., 2016). In contrast, the Hungarian ST8 strains developed just one favorable mutation (in position *grlA* S80) and proved unable to disseminate in the healthcare setting (Horvath et al., 2012; Fuzi, 2016). Similarly, while the ST22 strains carrying the double-serine mutations disseminated extensively in Western countries (Grundmann et al., 2014) some ST22 isolates in China that failed to develop these genetic traits showed lower incidence in the healthcare setting (Zhao et al., 2023).

### 4.2 Multidrug-resistant *Klebsiella pneumoniae*

The clonal shift with HA-MRSA was followed by a conspicuous clonal reduction observed with ESBL-producing *K. pneumoniae* in Hungary (Damjanova et al., 2006; Damjanova et al., 2008). Interestingly, in contrast to the previous polyclonal scene, all of the novel strains belonged to just three major STs (ST11, ST15, ST147) and carried the CTX-M-15 ESBL gene (Damjanova et al., 2006; Damjanova et al., 2008). The new major clones disseminated exclusively in adult hospital wards where fluoroquinolones were in extensive use but not in the perinatal intensive care units where these antibiotics were not considered an appropriate choice of therapy (Szilagyi et al., 2010). Interestingly in the perinatal intensive care units the MDR *K. pneumoniae* isolates remained polyclonal and primarily produced SHV type ESBL enzymes (Damjanova et al., 2008; Szilagyi et al., 2010) (Table S1). Similarly to MRSA the observed clonal change was associated with a rise in the incidence for ESBL-producing *K. pneumoniae* in Hungary (Szilagyi et al., 2010) (Table S2).

With regard to previous findings obtained with MRSA (Horvath et al., 2012) we performed propagation assays with major and minor ST ESBL-producing and fluoroquinolone resistant strains of *K. pneumoniae*. We found that the major clone/ST strains displayed significantly faster growth rates than minor clone isolates (Toth et al., 2014). In addition, some of our minor clone *Klebsiella* strains, originally susceptible to fluoroquinolones, suffered substantial fitness cost during *in vitro* induction of resistance to ciprofloxacin while other minor lineage strains ab ovo failed to assume high-level resistance (Toth et al., 2014). In contrast major ST MDR strains retained growth rates just slightly compromised relative to that of fluoroquinolone susceptible isolates (Toth et al., 2014) (Table 2).

All major clone MDR *K. pneumoniae* strains carried three QRDR mutations, including the double-serine alterations, in the *gyrA* S83, D87 and *parC* S80 positions. In contrast, the minor ST strains either failed to evolve any of these mutations or acquired fewer of them (Toth et al., 2014) (Table 1). These findings could account for the clonal dynamics observed with ESBL-producing *K. pneumoniae* in Hungary. Moreover, they may clarify the background of the widespread international dissemination of the CTX-M-15 enzyme.

Subsequently two additional major international lineages of MDR *K. pneumoniae* emerged and disseminated extensively worldwide. The ST258 clone, a close relative of the ST11 lineage, that played a significant role in the global spread of KPC-type carbapenemases and the ST307 clone (Bowers et al., 2015; Peirano et al., 2020) (Table S1). The available data shows that the carriage of QRDR mutations affecting primarily the double-serine (*gyrA* S83, *parC* S80) positions, but sometimes also an additional alteration in the *gyrA* D87 position, is a general feature of ST11, ST15, ST147; ST101, ST258 and ST307 MDR *K. pneumoniae* lineages (Bowers et al., 2015; Park et al., 2015; Roe et al., 2019; Zeng et al., 2020; Peirano et al., 2020; Núñez-Samudio et al., 2022; Cai and Wang, 2022; Rodrigues et al., 2022; Rodrigues et al., 2023) (Table 1). Accordingly, these strains command an energy benefit when acquiring an MDR phenotype advancing their dissemination in a fluoroquinolone environment allowing for the replacement of minor clone isolates (Damjanova et al., 2008; Chen et al., 2014; Wang Q. et al., 2024; Chatzidimitriou et al., 2024; Peirano and Pitout, 2025) (Table S1).

Moreover, similarly to HA-MRSA multiple epidemiological studies demonstrated that besides *β*-lactam antibiotics, the consumption of fluoroquinolons influences the incidence of ESBL-producing *K. pneumoniae* that mostly involves the rates of strains from major STs (Szilagyi et al., 2010; Forde et al., 2017; Wang et al., 2019; Corcione et al., 2021; Tan et al., 2022) (Table S2). In addition, Logan et al. (2019) observed that in pediatric wards, which are supposed to be low-fluoroquinolone areas the incidence of minor ST strains of *K. pneumoniae* producing carbapenemases was higher than that of major ST isolates. Authors emphasized that the findings are in contrast with that reported by multiple groups for adult departments.

### 4.3 Multidrug-resistant *Escherichia coli*

It is well-established that strains of both global lineages of MDR *E. coli*, the ST131 H30 and ST1193 clones, carry, among others, the energetically favorable double-serine QRDR mutations affecting the positions *gyrA* S83 and *parC* S80 (Colpan et al., 2013; Pitout and Finn, 2020; Pitout et al., 2022). Moreover, an emerging clone – ST410 – may be on the way of becoming another major international MDR lineage of *E. coli* (Pitout et al., 2023). ST410 isolates also characteristically carry the favorable double-serine mutations besides two additional alterations (Roer et al., 2018) (Table 1). Pitout et al. (2024) demonstrated that the novel clades of ST410, in contrast to previous ones, acquired the favorable QRDR mutations relatively recently and that might have contributed to the clone’s success with an MDR phenotype.

American scientists experimentally demonstrated a positive fitness effect associated with resistance to fluoroquinolones, similarly to that observed with the international STs of MRSA and MDR *K. pneumoniae*, in the global *E. coli* MDR lineage of ST131 H30 (Johnson et al., 2015a; Johnson et al., 2015b) (Table 2). This “fitness advantage” was linked to a number of favorable mutations in the *gyrA*, *parC*, and *parE* genes and a significantly weaker efflux activity relative to isolates from other lineages (Johnson et al., 2015a; Johnson et al., 2015b). This fitness benefit should have contributed to the extraordinary success of the ST131 H30 lineage and that of other major MDR clones of *E coli* (Nicolas-Chanoine et al., 2008, 2014; Price et al., 2013; Stoesser et al., 2016; Tchesnokova et al., 2019; Pitout and Finn, 2020; Pitout et al., 2022, 2024) (Table S1).

Moreover, subsequently Stoesser et al. (2016) analyzing ST131 H30 strains with WGS technique concluded that “strong selection pressure exerted by the widespread use of fluoroquinolones and extended-spectrum cephalosporins”…“most likely” played a major role in the selection of the H30 and H30R lineages of *E. coli* (Table S2). In addition, several groups suggested that there was an association between the success of the ST131 MDR lineage and fluoroquinolone resistance (QRDR mutations). Van der Donk et al. (2013) showed that the increased use of fluoroquinolones resulted in a rise in the incidence of ST131 strains. Banerjee and Johnson (2013) suggested that fluoroquinolone use was associated with the spread of ST131 *E. coli*. Li et al. (2021) linked the dissemination of the ST131 MDR clone to DNA gyrase and topoisomerase IV mutations. Cummins et al. (2021) showed that there was a link between resistance to fluoroquinolones, increased fitness and the prevalence of ST131-H30 and ST1193 isolates (Table S2).

Moreover, the fitness benefit commanded by the ST131 H30 and ST1193 clones might have contributed not only to a rise in the incidence of infections caused by these bacteria but also to the successful enteric colonization of healthy individuals by these lineages and by strains from other STs carrying one or two favorable QRDR mutations (Tchesnokova et al., 2023; Fuzi and Sokurenko, 2023).

### 4.4 Multidrug-resistant *Clostridioides difficile*

In *C. difficile* and some other species—*P. aeruginosa, B. cepacia, C. jejuni*—the residue playing a prominent role in the binding of fluoroquinolones to DNA gyrase (position *gyrA* 83 in *E. coli*) is not serine but threonine. Moreover, *C. difficile,* like some additional slow-growing bacteria — *C. jejuni* and the *Mycobacteria* — are void of the topoisomerase IV enzyme. Consequently QRDR resistance in these species will primarily reside with alterations in the DNA gyrase gene (Dridi et al., 2002).

The most renowned international MDR lineage of *C. difficle* is the 027 ribotype. Ribotype 027 isolates are characteristically resistant to fluoroquinolones and carry the *gyrA* T82I mutation (He et al., 2013; Spigaglia, 2016) (Table 1). Wasels et al. (2015) reported that resistance to fluoroquinolones in ribotype 027 strains was associated with a very slight fitness cost which was linked to the presence of the *gyrA* T82I mutation. However, Vernon et al. (2019) subsequently unequivocally demonstrated that the *gyrA* T82I mutation confers a fitness advantage on many 027 isolates in a strain-specific fashion (Table 2). Some additional DNA gyrase mutations were also associated with greater fitness in *C. difficile*, however, these alterations conferred significantly lower MIC values to fluoroquinolones than the *gyrA* T82I mutation (Vernon et al., 2019).

These findings strongly argue for a mechanism similar to that observed with HA-MRSA, ESBL-producing *K. pneumoniae* and MDR *E. coli* since mutations affecting the *gyrA* T82 position have also been shown to be a feature of isolates of additional major-non-027-international ribotypes of *C. difficile* (Carman et al., 2009; Saxton et al., 2009; Spigaglia et al., 2010; Lin et al., 2011; Solomon et al., 2011; Lee et al., 2014; Baldan et al., 2015; Kuwata et al., 2015; Spigaglia, 2016; Imwattana et al., 2021; Shu et al., 2023; Plankaova et al., 2023) (Table 1).

In contrasts to the rather restricted clonal landscape observed with HA-MRSA, ESBL-producing *K. pneumoniae* and MDR *E. coli* where the capacity of developing favorable *gyrA* and *parC* mutations seems to be restricted to just a few international ST strains (see above), multiple lineages of *C. difficile* proved capable of evolving the *gyrA* T82I mutation which resulted in a relative diversity in the clonal spectrum (Bauer et al., 2011; Tickler et al., 2014; Freeman et al., 2015; Liu et al., 2023) (Table 1).

The extraordinary expansion of the 027 ribotype and that of multiple additional *gyrA* T82I-positive lineages (Goorhuis et al., 2008; Freeman et al., 2015; Liu et al., 2023) (Table S1) plus a well-documented clonal replacement attest to the superior dissemination capacity of international *C. difficle* clones in a fluroquinolone environment.

A comprehensively investigated clonal shift with *C. difficile* was reported from a Korean hospital. The previously prevalent ribotype 001 strains were replaced by isolates from the 014, 017, and 018 ribotypes within a couple of years (Lee et al., 2014) (Table S1). Interestingly, all of the novel ribotype strains carried the energetically favorable T82I *gyrA* mutation while the Korean ribotype 001 isolates harbored a different mutation in the same position (T82V *gyrA*) which was previously demonstrated to be associated with a high fitness cost (Wasels et al., 2015).

Moreover, the proposed “fluoroquinolone mechanism” for the selection of the major clones of *C. difficile* is supported by the observation that the proportion of ribotype 027 strains is significantly greater in adult wards than in pediatric units (McFarland et al., 2016). Furthermore, an abundance of studies demonstrated that a decrease in the consumption of fluoroquinolones results in a decline in the incidence of *C. difficile* infections in general and of those caused by major clone (ribotype) strains in particular (Pepin et al., 2004; McDonald et al., 2005; LeBlanc et al., 2006; Sarma et al., 2015; Dingle et al., 2017; Rizzo et al., 2019; Redmond et al., 2019; Kazakova et al., 2020; Guh et al., 2020; Slimings and Riley, 2021; Couture et al., 2024) (Table S2).

### 4.5 Multidrug-resistant *Enterococcus faecium*

MDR *E. faecium* showing resistance to various antibiotics including vancomycin (VRE) poses a serious challenge for antibiotic therapy worldwide. A few lineages related to a single international clonal complex of VRE (CC17) dominate the hospital setting in many geographic areas (Willems et al., 2005; Freitas et al., 2016).

VRE disseminated in Europe in the 1990s initially as an enteric colonizer due to the extensive use of avo*parc*in, a veterinary glycopeptide antibiotic (Van den Bogaard et al., 1997). However, no major clone of VRE was observed in the healthcare setting during this period (Clark et al., 1993; Van den Braak et al., 1998). Then surprisingly major MDR lineages of VRE all linked to a single clonal complex, CC17, started to expand across large geographic areas (Willems et al., 2005; Cattoir and Leclercq, 2013) (Table S1). Most of these strains were characteristically resistant to fluoroquinolones and carried – sometimes among others – the double-serine QRDR alterations (Leavis et al., 2006; López et al., 2011; Valdezate et al., 2012; Troscianczyk et al., 2022) (Table 1). Moreover, the use of fluoroquinolones was reported to be associated with the incidence of strains from the CC17 VRE lineage (Top et al., 2007). Furthermore, fluoroquinolone consumption was observed to be more closely associated with VRE rates than the consumption of glycopeptide antibiotics (Forstner et al., 2015) (Table S2). In addition, CC17 strains were demonstrated to start to extensively disseminate when having acquired resistance to fluoroquinolones by evolving the favorable QRDR mutations and only subsequently developed resistance to vancomycin (López et al., 2011; Cattoir and Leclercq, 2013). Moreover, this clone was recently reported to spread as a vancomycin-susceptible but fluoroquinolone-resistant lineage featuring the double-serine mutations (Aung et al., 2023) (Table S1). Finally, it is certainly not accidental that all MDR (VRE) *E faecium* lineages are associated with a single clonal complex: it was demonstrated that the capacity to evolve the double-serine QRDR mutations is extremely rare among *E. faecium* isolates (De Lastours et al., 2017).

### 4.6 Multidrug-resistant *Pseudomonas aeruginosa*

The dissemination of MDR *P. aeruginosa*, similarly to many other resistant pathogens, is well-established to be clonal. Besides the three main international STs – ST235, ST111 and ST175 (Cabot et al., 2012; Del Barrio-Tofiño et al., 2017; Pérez et al., 2019; Recio et al., 2021; Torrens et al., 2022) (Table S1)—multiple local high-risk clones were also observed (Pérez et al., 2019; Kocsis et al., 2019; Silveira et al., 2020; Torrens et al., 2022; Hu et al., 2021; Kiyaga et al., 2022).

The carriage of the energetically favorable double QRDR mutations—*gyrA* T83I and *parC* S87L in *P. aeruginosa*—is a characteristic feature of all three major international MDR clones of ST235, ST111 and ST175 (Cabot et al., 2012; Kos et al., 2015; Recio et al., 2021; Torrens et al., 2022) (Table 1). Moreover, the ST175 strains usually carry an additional *gyrA* D87N QRDR alteration (Cabot et al., 2012; Kos et al., 2015; Del Barrio-Tofiño et al., 2017; Torrens et al., 2022).

Some of the local high-risk clones (ST773, ST654; ST357, ST463) were also reported to often carry the favorable double-threonine/serine QRDR mutations (Kocsis et al., 2019; Torrens et al., 2022; Kiyaga et al., 2022; Zhang et al., 2023; Jung et al., 2024). However, many minor clone strains were observed to harbor just one of the energetically favorable mutations which is often combined with additional, diverse, less common DNA gyrase and topoisomerase IV alterations (Kos et al., 2015; Del Barrio-Tofiño et al., 2017; Kiyaga et al., 2022).

The significance of the double threonine/serine QRDR mutations in *P. aeruginosa* was demonstrated by Treepong et al. (2018) who showed that the epidemiological success of the ST235 clone commenced when the strains assumed resistance to fluoroquinolones which was only followed by the acquisition of an MDR phenotype. Moreover, Brüggemann et al. (2018) demonstrated that the ST235 strains of *P. aeruginosa*, despite a shared clonal ancestry, “possessed individual evolutionary histories” when having acquired an MDR phenotype. This observation raises the question: if not the genetically related evolution, then what is the common feature in the successful isolates of the ST that permitted/facilitated dissemination? The answer seems obvious, the carriage of the double-threonine/serine QRDR mutations. Furthermore, Hakki et al. (2019) demonstrated that the use of fluoroquinolones results in an increase in the rate of MDR ST111 *P. aeruginosa* with strains displaying resistance to antibiotics the patients were not exposed to (Table S2).

### 4.7 Multidrug-resistant *Acinetobacter baumannii*

The dissemination of MDR *A. baumannii* – similarly to other MDR pathogens – remains largely clonal. Strains from a few global clonal complexes (“international clones”) comprising multiple STs are responsible for the majority of infections (Karah et al., 2012; Hamidian and Nigro, 2019; Valcek et al., 2022; Castillo-Ramírez, 2023) (Table S1). The major ST MDR strains characteristically carry double-serine QRDR mutations affecting positions: *gyrA* S81 and *parC* S80 (Warner et al., 2016; Shen et al., 2016; Nodari et al., 2020; Wiradiputra et al., 2023) (Table 1) which are equivalent to those observed in the global clones of other international MDR lineages.

The crucial role of fluoroquinolone use in the dissemination of MDR *A. baumannii* was confirmed by Matsui et al. (2018) conducting a large epidemiological study in Japan. The authors concluded that the judicious use of “…fluoroquinolones may hold the key to overcoming epidemic clones” of MDR *A. baumannii*.

## 5 Not-clone-specific positive fitness schemes associated with MDR phenotype

Besides the favorable double-serine QRDR fitness effect MDR pathogens command/employ a variety of not-clone-specific energy saving/generating schemes to support the carriage and expression of an extra antibiotic resistance gene cargo which will not compromise antibiotic resistance.

### 5.1 Genome size and fitness

It has been suggested more than fifteen years ago that the size of the bacterial genome impacts the capacity of a particular pathogen to assume an MDR phenotype (Projan, 2007). Bacterial species with large genomes seem capable of harboring a greater cargo of antibiotic resistance genes without suffering serious fitness cost than species with smaller genomes (Projan, 2007; Iranzo et al., 2017). A newly acquired excess block of genes will constitute a much smaller proportion in a large bacterial genome than in a small bacterial genome and will confer a smaller fitness cost onto the agent (Iranzo et al., 2017).

It is certainly not accidental that the pathogens comprising the major international MDR clones mentioned above command the biggest genomes among human pathogenic bacteria (Projan, 2007). In addition, it should not be a contingency that human pathogenic bacteria featuring the largest genomes, *P. aeruginosa* and *K. pneumoniae* (Projan, 2007), are well-known to be capable of acquiring a pandrug-resistant phenotype (Kryzhevskyi et al., 2023; de Man et al., 2016). Interestingly, *A. baumannii* featuring a somewhat smaller genome (Duan et al., 2024) was also observed to assume pandrug-resistance (Fernández-Vázquez et al., 2023). However, this exploit apart from the considerable size of the genome, could be the consequence of some additional energy saving schemes in *A. baumannii*: a highly sophisticated management of plasmids (Maslova et al., 2022), the application of special metabolic pathways (Djahanschiri et al., 2022) and, most importantly, an unique technique for the judicious control of its integrase enzymes (Couvé-Deacon et al., 2019) (the latter see in detail in chapter: Plasmids and integrons). Interestingly, *E. coli* and *Salmonella* spp. which command somewhat bigger genomes than *A. baumannii* but smaller than those of *P. aeruginosa* and *K. pneumoniae* (Projan, 2007) have not been reported to have acquired pandrug-resistance to date hinting at the relevance of some unique energy saving devices observed in *A. baumannii*.

*S. aureus* and *E. faecium* command smaller genomes than any of the gram-negative pathogens mentioned above (Projan, 2007). Accordingly, pandrug-resistance has not been observed in these gram-positive agents. Moreover, serious limitations remain even as to the MDR phenotypes that *S. aureus* and *E. faecium* can assume. *S. aureus* was demonstrated to suffer highly significant fitness cost when acquiring resistance to both daptomycin and vancomycin (Roch et al., 2017; McGuinness et al., 2017). Furthermore, vancomycin resistant *S. aureus* (VRSA) strains usually remain susceptible to a variety of non-glycopeptide antibiotics (Cong et al., 2019) which is most certainly a fitness effect. In addition, the incidence of VRSA remains very low despite several reports of individual infections occurring across large geographical areas (McGuinness et al., 2017). The failure of VRSA to disseminate is certainly linked to a serious compromise in fitness (McGuinness et al., 2017). Moreover, similarly to *S. aureus*, resistance to daptomycin results in a serious fitness cost in vancomycin resistant *E. faecium* (VRE) that compromises even the strains’ resistance to glycopeptides (Zeng et al., 2022).

### 5.2 Plasmids and integrons

Bacteria evolved several well-defined and not clone-specific energy-saving/generating schemes – independent of the size of genome—which assist in the preservation of fitness and allow proper growth rates subsequent to the acquisition of extra gene cargoes without seriously compromising antibiotic resistance.

One of the most important vehicles bacteria employ in their defensive strategy against exposure to harmful substances, including antibiotics, are plasmids. Plasmids allow bacteria to transiently acquire helpful genes that can be subsequently eliminated when conditions rehabilitate permitting for the restoration of fitness. The carriage of plasmids is associated with fitness cost (Modi and Adams, 1991; Adler et al., 2014; Dunn et al., 2021) that is primarily related to the metabolic cost and the molecular conflicts emanating from the “interactions of the plasmid and host machineries” (Modi and Adams, 1991).

However, the plasmid-related fitness cost can subsequently be attenuated mainly by the evolution of compensatory mutations (Yano et al., 2016; Hall et al., 2021; Ares-Arroyo et al., 2022; Carrilero et al., 2023). Moreover, some plasmids were reported to exert just a minimal adverse effect onto host bacteria. The fitness effects of some plasmids were thoroughly investigated in the major MDR clones of *E. coli* and were established to be usually minimal rendering their carriage economical (San Millan and MacLean, 2017; Johnson et al., 2019; Dunn et al., 2021; Palkovicova et al., 2022; Vanacker et al., 2023; Chen et al., 2024).

The main source of fitness cost associated with the carriage of plasmids was demonstrated to be the number of genes expressed (Sandegren et al., 2012; Adler et al., 2014; Vogwill and MacLean, 2015; Wein et al., 2019; Vanacker et al., 2023) although the energy loss proved very diverse across individual genes (Rajer and Sandegren, 2022). However, it is not just the expression of genes but also the level of expression that is associated with fitness cost during both plasmid-, and chromosomal adaptation (Kloos et al., 2021). The extensive dissemination of the IncP-1 plasmid in a variety of bacterial species was linked to its sophisticated machinery capable of decreasing (“fine-tuning”) gene expression and minimize fitness cost (Adamczyk and Jagura-Burdzy, 2003). The successful horizontal spread of the *mcr-1* colistin resistance gene was similarly established to have been permitted by the scaling back (“optimization”) of its expression (Ogunlana et al., 2023). Moreover, the substantial decrease of transcription in multiple genes of the broad-host range plasmid pBP136 was observed to “drastically” improve its maintenance in host bacteria (Elg et al., 2024).

Consequently, the optimization/fine-tuning of the level of gene expression in both the chromosome and plasmids is a salient fitness maintaining exercise for bacteria. The ability to collectively control and optimize the expression of multiple genes, including many of those associated with resistance to antibiotics, bacteria employ the genetic elements called integrons. Integrons are ubiquitos in bacteria and can be located on both plasmids and the chromosome (Noel et al., 2022). Various genes recruited to an integron gene cassette have a common promoter which allows for maintaining a collective level of expression. Integron genes with strong promoters will be expressed usually at a much higher level than integron genes with weak promoters (Fonseca and Vicente, 2022). Strains of the major lineages of MDR *E. coli* (ST131 H30; ST1193) harbor mostly class 1 integrons (Li et al., 2020; Li et al., 2021) featuring generally weak promoters (Vinué et al., 2011; Wei et al., 2013) allowing for a considerable economization.

Some data are available on the promoter types of integrons in major MDR clone isolates in some additional pathogens. The carriage of type 1 integrons was reported common also in MDR strains of *K. pneumoniae* (Derakhshan et al., 2016; Bakr and Zaki, 2019; Wang et al., 2023). Moreover, Wang et al. (2023) detected mostly the “relative weak” PcH1 promoter in the integrons of their MDR *K. pneumoniae* isolates. Weak promoters remain dominant also in class 1 integrons in MDR *Proteus* spp. and were demonstrated to be associated with a favorable fitness effect (Xiao et al., 2019). Moreover, similarly to MDR *E. coli* and MDR *K. pneumoniae* it was recently established that the carriage of class 1 integrons is characteristic for MDR high-risk clones of *P. aeruginosa* (Lee et al., 2023). The relevance of energy conservation by the judicious expression of integron genes in *P. aeruginosa* was recently demonstrated by Kikuchi et al. (2024) who showed that a novel integron acquired years earlier by the species had originally harbored a strong promoter but started to globally disseminate only after its expression has been attenuated.

The application of weak promoters is not the only means for saving energy with integron function. *A. baumannii* uses an exceptional mechanism for the control of its costly integrase enzymes. Integrons are associated with the integrase enzyme, a crucial element of the integron system, catalyzing both insertion and excision of gene cassettes. The expression of the integrase must be strictly controlled to prevent substantial DNA damage, a consequence of inadvertant activity (Cambray et al., 2011). Most bacteria restrict the unintended expression of integrase by employing the LexA SOS system (Cambray et al., 2011). However, this type of integrase control was demonstrated in *E. coli* to result in an enormous fitness cost especially if the integrase has to be regularly blocked by an SOS response due to steady stress effects like continuous exposure to antibiotics (Kozuch et al., 2020; Jones and Uphoff, 2021). Moreover, MDR bacteria often carry multiple integrons whose integrases must be kept in check individually to prevent substantial damage to the genetic makeup (Couvé-Deacon et al., 2019; Xiu et al., 2024).

*A. baumannii* lack the LexA system and use instead the “umuDAb” protein for stress control (Aranda et al., 2013; Gregg-Jolly, 2022) that was reported to facilitate the acquisition of some antibiotic resistance determinants (Norton et al., 2013). However, *A. baumannii* basically employs a quite different, and highly energy conserving strategy for the control of its integrase enzymes: it uses special integron promoters which will ab ovo restrict integrase activity (Couvé-Deacon et al., 2019; Fonseca and Vicente, 2022; Xiu et al., 2024). Integrons in *A. baumannii* usually carry structurally strong casette promoters which are attenuated, however, will exert sufficient transcriptional interference with the integrase promoter to limit the expression of the enzyme and conserving thereby substantial energy (Couvé-Deacon et al., 2019; Xiu et al., 2024). This unique mechanism of integrase control might have contributed to the ability of *A. baumannii* to acquire a pandrug-resistant phenotype with a relatively modest size of genome compared with those of *P. aeruginosa and K. pneumoniae*.

Energy conservation by the “silencing” of integrase genes was observed also in the international MDR clones of *E. coli* (ST131, ST1193). In these lineages a considerable proportion of the integrons were reported to have been truncated and the deletions mostly affected the integrase genes preventing the enzyme’s transcription (Li et al., 2021; Wyrsch et al., 2022).

Furthermore, as mentioned above, *A. baumannii* commands some additional specific energy generating/saving schemes which besides the restriction of integrase transcription should support the carriage of excess gene cargoes: the employment of some special metabolic pathways (Djahanschiri et al., 2022) and the highly efficient conduct of the plasmidome (Maslova et al., 2022).

Besides compensatory mutations, the fitness cost associated with plasmids can be further mitigated by the transfer of long-standing antibiotic resistance genes from the plasmid to the chromosome which also results in some fitness advantage that may support the maintenance of an MDR phenotype. Plasmid genes which come from an alien agent command a codon-usage scheme that is different from that of the host bacterium to which the tRNA gene pool of the cell has been tailored (López et al., 2019; López et al., 2020). However, plasmid genes transferred to the chromosome will adapt with time and gradually assume the codon usage scheme of the host which should be associated with some energy conservation (López et al., 2020). It is certainly not accidental that the transfer of antibiotic resistance genes from plasmids to the chromosome was observed in some major MDR clone strains of *E. coli* (Price et al., 2013), *K. pneumoniae* (Mathers et al., 2017); *P. aeruginosa* (Hong et al., 2016; Papagiannitsis et al., 2020) and *A. baumannii* (Valcek et al., 2022; Wang W. et al., 2024). The gene transfer from plasmid to chromosome may involve the whole integron which will then be often further economized by the elimination of the integrase gene (Cury et al., 2016; Goswami et al., 2020).

## 6 Virulence, antibiotic resistance and fitness: always a trade-off

Although the fitness benefit/conservation conferred by the double serine QRDR mutations, low cost plasmids, integrons with weak promoters and other fitness schemes permit the international MDR bacteria to carry an extensive extra gene cargo, the energy gain has its limits. As we have seen with *S. aureus* and *E. faecium*, sometimes less relevant antibiotic resistance genes have to be sacrificed to maintain appropriate fitness with the carriage of an indispensible resistance gene cargo (Roch et al., 2017; McGuinness et al., 2017; Zeng et al., 2022).

In addition, the “fitness limit” warrants that virulence genes, which are not essential for survival in the antibiotic environment of the healthcare setting, will often be deleted in a trade-off in the major MDR clones of bacteria. The “fluoroquinolone-related” major MDR clone strains are usually less virulent than the antibiotic susceptible isolates from the same species and it is most certainly the command of appropriate fitness that primarily supports their dissemination in the healthcare setting.

Martínez and Baquero (2002) emphasized more than twenty years ago the precedence of antibiotic resistance over virulence in the selection of international clones in their classic paper. They observed: “If antibiotics are almost ubiquitously present in the hosts, as in an intensive care unit…the spread of the resistant bacteria is favored”.

We previously reviewed the virulence features of MDR clones in *S. aureus, E. coli, K. pneumoniae, E. faecium, C. difficile* and *P. aeruginosa* (Fuzi et al., 2020). Although some virulence factors – primarily the formation of biofilm – contribute to the dissemination of multiple international MDR clones we showed that these pathogens usually harbor fewer virulence determinants than more susceptible lineages from the same species (Fuzi et al., 2020). In the following we are going to review some well-documented examples demonstrating that the carriage of virulence features is often a function of available fitness subsequent to the acquisition of an indispensible antibiotic resistance gene cargo.

Strains from the major MDR ST8 clone of HA-MRSA were reported to carry fewer virulence factors than more susceptible HA-MRSA isolates (Dauwalder et al., 2008). Moreover, the SCCmec II, III elements which are typically harbored by HA-MRSA strains and are bigger and confer significantly higher MIC values to *β*-lactam antibiotics than the SCCmec IV/V casettes often include a mobile genetic element (*psm-mec*) whose product compromises the accessory gene regulator system (*agr*) attenuating the virulence of HA-MRSA isolates (Kaito et al., 2013; McCarthy et al., 2015).

Moreover, Duprilot et al. (2020) showed that ST131 strains of MDR *E. coli* sacrificed multiple virulence genes to retain appropriate fitness that should have contributed to the clone’s success. The ST410 clone of *E. coli* shows a relatively low virulence-associated factor score and has started to emerge as a major international MDR lineage not after acquiring more virulence genes but only subsequent to having evolved the double-serine mutations plus two additional QRDR alterations (Pitout et al., 2024). This set of mutations should have conferred both the extra fitness benefit and the appropriate level of fluoroquinolone resistance that was required for dissemination in adult hospital wards.

The attenuation of virulence in some of the so-called hypervirulent MDR major STs of *K. pneumoniae* is well-reflected in the observation that the plasmid responsible for the introduction of multiple virulence genes into many of these isolates proved capable of dissemination exclusively after having significantly lowered the associated fitness cost by eliminating the relevant virulence gene cluster of *iroBCDN* (Jia et al., 2024).

Dingle et al. (2023) demonstrated that β-lactam resistance conferred by PBP mutations were just as characteristic for 027 strains of *C. difficile* as the carriage of the energetically favorable *gyrA* T82I and *gyrB* D426N/V alterations. In addition, they observed that two fluoroquinolone susceptible lineages commanding similar PBP mutations (010 and 039) proved quite successful in non-fluoroquinolone environments. Interestingly the 010 and 039 lineages are both non-toxigenic which might have conferred a fitness benefit onto the isolates. Since PBP mutations were demonstrated to have been associated with fitness cost in *Clostridium perfringens* (Park and Rafii, 2017), Dingle et al.’s (2023) observation may suggest that the *gyrA* T82I and *gyrB* D426N/V mutations might have conferred a fitness benefit onto the 027 strains that could have permitted the clone to collectively acquire PBP mutations and the toxin gene cargo. In contrast the 010 and 039 lineages proved unable to evolve the favorable QRDR mutations that might have prevented these clones from carrying both the mutated PBP proteins and an extra toxin gene load.

Though MDR strains of *P. aeruginosa* often display an attenuated virulence profile relative to susceptible isolates (Peña et al., 2015) many high-risk clone strains from the sequence types: ST235; ST111; ST773; ST357 were observed to show considerable virulence which was predominantly linked to the production of the exoU toxin (Peña et al., 2015; Sánchez-Diener et al., 2017; Papagiannitsis et al., 2020; Recio et al., 2021; Kiyaga et al., 2022; Stoikov et al., 2023; Wu et al., 2024; Jung et al., 2024). Interestingly, in contrast to these clones, strains from the ST175 MDR major lineage, one of the biggest MDR groups in *P. aeruginosa*, are mostly less virulent and remain void of the *exoU* gene (Sánchez-Diener et al., 2017; Recio et al., 2021; Silva et al., 2023). The question arises, why are many high-risk clones of *P. aeruginosa* capable of harboring a greater virulence gene cargo, involving primarily the exoU toxin-related genes, than ST175 strains?

The exoU+ TTSS type III genetic background was demonstrated to be conducive for the evolution of QRDR mutations in general and for the development of the threonine/serine alterations in particular in *P. aeruginosa* (Agnello and Wong-Beringer, 2012). Most of the virulent high-risk clone MDR strains characteristically carry the double threonine/serine QRDR mutations (Cabot et al., 2012; Kos et al., 2015; Recio et al., 2021; Torrens et al., 2022) conferring a fitness advantage onto the isolates. Alternatively, the ST175 strains are well-established to typically carry an additional *gyrA* D87 substitution (Cabot et al., 2012; Kos et al., 2015; Recio et al., 2021; Torrens et al., 2022) that should compromise the fitness gain associated with the double-threonine/serine mutations and prevent the carriage of a greater virulence gene cargo, like exoU. The mutations affecting an equivalent position to *gyrA* D87 in *P. aeruginosa* were demonstrated to be associated with a fitness cost in various *Enterobacterales* spp. by several groups of investigators (Komp Lindgren et al., 2005; Marcusson et al., 2009; Baker et al., 2013).

Finally, in contrast to other pathogens, major clone strains of MDR *A. baumannii* can often display greater virulence than minor clone isolates (Kumkar et al., 2022; Park et al., 2023). This interesting characteristic of the MDR major clone strains is most certainly permitted by the specific energy generating/saving schemes commanded by *A. baumannii*, primarily the efficient control of its integrase enzymes (Couvé-Deacon et al., 2019; Xiu et al., 2024) that may also allow for the acquisition of pandrug-resistance with a relatively modest size of genome.

## 7 Perspective

Several lines of strong circumstantial evidence suggest that bacteria need extra energy when they acquire an excess gene cargo to be able to cope with the surplus load. However, enhancement of the basic energy generating metabolic activity is well-established to increase susceptibility to antibiotics. Accordingly bacterial pathogens need to command/employ other energy generating/saving schemes that will confer a fitness benefit without compromising susceptibility. Some QRDR mutations associated with fitness benefit have been demonstrated to support the acquisition and expression of antibiotic resistance genes contributing substantially to the dissemination of the major international MDR clones in various bacterial species (Fuzi et al., 2017; Fuzi and Sokurenko, 2023). A large genome also promotes the physiologically sustainable carriage of acquired antibiotic resistance genes rendering some bacterial species more prone to assume MDR phenotypes (Projan, 2007). Moreover, additional general and species-specific energy saving/generating schemes contribute to the ability of pathogens to acquire diverse quantities of excess gene cargoes (Table 3). Bacteria commanding more abundant/efficient fitness schemes will prove more capable of assuming an MDR phenotype and/or retaining virulence genes.

The fitness schemes reviewed in this paper are certainly relevant for MDR bacteria, however, comprise only a fraction of the miscellaneous techniques pathogens employ to save/generate extra energy without compromising antibiotic resistance. The impact of the efficient management of various mobile genetic elements, phages, ncRNAs and the effect of distinct metabolic pathways – among others – remain to be investigated. Accordingly, future studies should be aimed at exploring additional beneficial fitness schemes assisting the acquisition of an MDR phenotype and applying a fitness approach to any research associated with antibiotic resistance.

## Data Availability

Publicly available datasets were analyzed in this study. This data can be found at PubMed.
